# Update: Influenza Activity — United States and Worldwide, May 20–October 13, 2018

**DOI:** 10.15585/mmwr.mm6742a3

**Published:** 2018-10-26

**Authors:** Eric J. Chow, C. Todd Davis, Anwar Isa Abd Elal, Noreen Alabi, Eduardo Azziz-Baumgartner, John Barnes, Lenee Blanton, Lynnette Brammer, Alicia P. Budd, Erin Burns, William W. Davis, Vivien G. Dugan, Alicia M. Fry, Rebecca Garten, Lisa A. Grohskopf, Larisa Gubareva, Yunho Jang, Joyce Jones, Krista Kniss, Stephen Lindstrom, Desiree Mustaquim, Rachael Porter, Melissa Rolfes, Wendy Sessions, Calli Taylor, David E. Wentworth, Xiyan Xu, Natosha Zanders, Jacqueline Katz, Daniel Jernigan

**Affiliations:** ^1^Epidemic Intelligence Service, CDC; ^2^Influenza Division, National Center for Immunization and Respiratory Diseases, CDC.

During May 20–October 13, 2018,* low levels of influenza activity were reported in the United States, with a mix of influenza A and B viruses circulating. Seasonal influenza activity in the Southern Hemisphere was low overall, with influenza A(H1N1)pdm09 predominating in many regions. Antigenic testing of available influenza A and B viruses indicated that no significant antigenic drift in circulating viruses had emerged. In late September, the components for the 2019 Southern Hemisphere influenza vaccine were selected and included an incremental update to the A(H3N2) vaccine virus used in egg-based vaccine manufacturing; no change was recommended for the A(H3N2) component of cell-manufactured or recombinant influenza vaccines. Annual influenza vaccination is the best method for preventing influenza illness and its complications, and all persons aged ≥6 months who do not have contraindications should receive influenza vaccine, preferably before the onset of influenza circulation in their community, which often begins in October and peaks during December–February. Health care providers should offer vaccination by the end of October and should continue to recommend and administer influenza vaccine to previously unvaccinated patients throughout the 2018–19 influenza season (*1*). In addition, during May 20–October 13, a small number of nonhuman influenza “variant” virus infections^†^ were reported in the United States; most were associated with exposure to swine. Although limited human-to-human transmission might have occurred in one instance, no ongoing community transmission was identified. Vulnerable populations, especially young children and other persons at high risk for serious influenza complications, should avoid swine barns at agricultural fairs, or close contact with swine.^§^

## United States

**FIGURE 1:**
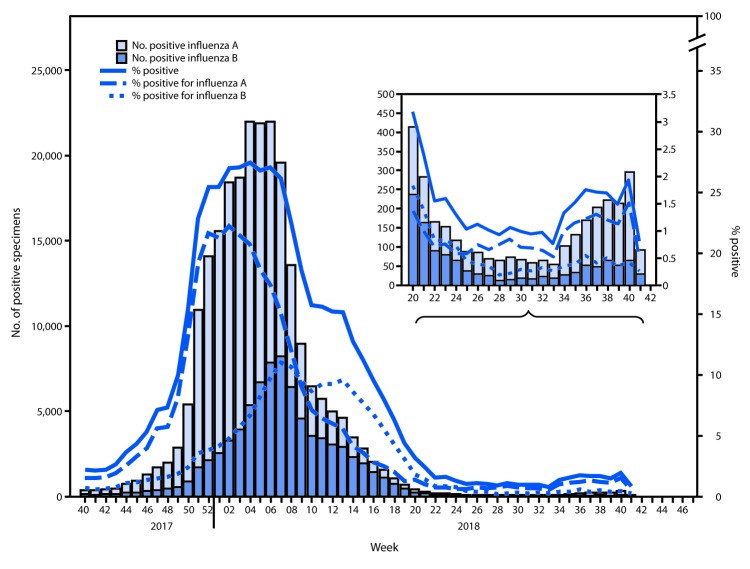
Number*and percentage of respiratory specimens testing positive for influenza reported by clinical laboratories, by influenza virus type and surveillance week — United States, October 1, 2017–October 13, 2018^†^ *A total of 238,440 (16.4%) of 1,452,986 tested were positive during October 1, 2017–October 13, 2018. ^†^ As of October 19, 2018.

**FIGURE 2:**
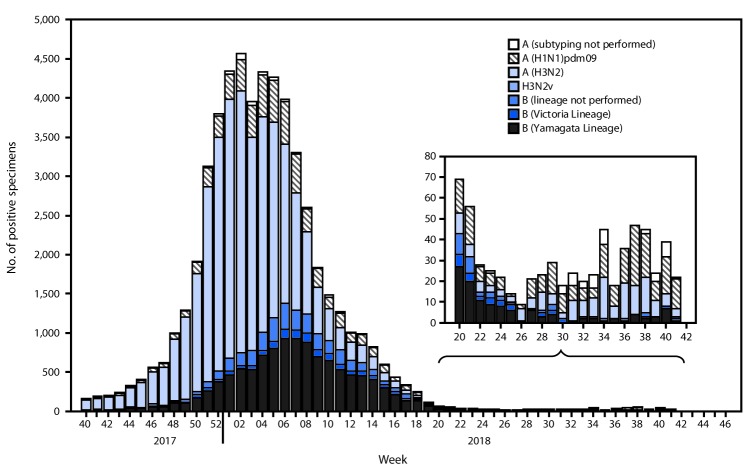
Number* of respiratory specimens testing positive for influenza reported by public health laboratories, by influenza virus type, subtype/lineage, and surveillance week — United States, October 1, 2017–October 13, 2018^†^ * N = 54,920. ^†^ As of October 19, 2018.

The U.S. influenza surveillance system^¶^ is a collaboration between CDC and federal, state, local, and territorial partners and uses eight data sources to collect influenza information, six of which operate year-round. During May 20–October 13, U.S. clinical laboratories tested 197,295 respiratory specimens for influenza, and 2,763 (1.4%) were positive (Figure 1), including 1,801 (65.2%) that were positive for influenza A viruses and 962 (34.8%) that were positive for influenza B viruses. Public health laboratories in the United States tested 5,863 respiratory specimens for influenza viruses; among these, 587 were positive for seasonal influenza viruses (Figure 2), including 442 (75.34%) positive for influenza A viruses and 145 (24.7%) for influenza B viruses. Influenza B viruses were more commonly detected than influenza A viruses from May until mid-June, whereas influenza A predominated from late June onward. A total of 400 (90.5%) of the seasonal influenza A viral specimens were subtyped by public health laboratories; among these, 233 (58.3%) were influenza A(H1N1)pdm09, and 167 (41.8%) were influenza A(H3N2). Of the 118 (81.4%) influenza B viruses for which lineage was determined, 94 (79.7%) belonged to the B/Yamagata lineage and 24 (20.3%) to the B/Victoria lineage. CDC received reports of a small number of influenza outbreaks during the summer, including domestic origin outbreaks along with influenza virus infection identified in returning international travelers.

During May 20–October 13, data obtained from the U.S. Outpatient Influenza-Like Illness Surveillance Network (ILINet) indicated that the weekly percentage of outpatient visits to health care providers for influenza-like illness (ILI)** remained below the national baseline^††^ of 2.2%, ranging from 0.6% to 1.4%. All regions remained below their region-specific ILI baselines. During the first 2 weeks of October, ILI activity levels^§§^ for all reporting jurisdictions were minimal and, although a small number of jurisdictions have reported the geographic spread of influenza activity^¶¶^ as local, approximately 60% of all reporting jurisdictions reported sporadic activity. Data from CDC’s National Center for Health Statistics Mortality Surveillance System indicated that the percentage of deaths attributed to pneumonia and influenza remained below the epidemic threshold*** during this period. Of the 183 influenza-associated pediatric deaths reported to CDC that occurred during the 2017–18 influenza season, five occurred during May 20–September 29. The first influenza-associated pediatric death occurring during the 2018–19 season was reported to CDC in mid-October. 

## Worldwide

CDC serves as the WHO Collaborating Center for Surveillance, Epidemiology, and Control of Influenza, one of six WHO Collaborating Centers for Influenza in the WHO Global Influenza Surveillance and Response System (GISRS).^†††^ CDC, along with other international public health partners, provides surveillance and virus characterization data to WHO. The timing of influenza activity and the predominant circulating virus around the world can vary by region.^§§§^ Overall, reported Southern Hemisphere influenza activity has been relatively low and fairly mild, with influenza A(H1N1)pdm09 viruses predominating in most regions.^¶¶¶^ Influenza data from GISRS during May 20–September 30 in temperate climate South American countries suggest that activity began to increase in mid-May and peaked in August. Influenza A(H3N2) predominated in Chile and Paraguay. In temperate Southern Africa, influenza activity increased in April and peaked in June, with A(H1N1)pdm09 predominating. A second wave of elevated activity in Southern Africa of mostly influenza B began in late August and peaked in September. Influenza activity in Australia and New Zealand was below seasonal threshold with A(H1N1)pdm09 predominating. Influenza activity in regions with more tropical climates (Central America and the Caribbean, tropical South America, Southern Asia, and Southeast Asia) was more variable, but A(H1N1)pdm09 virus predominated in most countries. Influenza A(H1N1)pdm09, A(H3N2), and B viruses cocirculated in Eastern Africa, and influenza A(H1N1)pdm09 and A(H3N2) viruses cocirculated in Southern Asia.

## Genetic and Antigenic Characterization of Influenza Viruses

The components for the Northern Hemisphere 2018–19 influenza vaccines were selected in February 2018, during one of the twice-yearly WHO-sponsored vaccine consultation meetings. The recommended Northern Hemisphere 2018–19 trivalent influenza vaccine composition included an A/Michigan/45/2015 (H1N1)pdm09-like virus, an A/Singapore/INFIMH-16–0019/2016 (H3N2)-like virus, and a B/Colorado/06/2017-like virus (B/Victoria lineage), with an additional influenza B virus (B/Phuket/3073/2013-like [B/Yamagata lineage]) recommended for quadrivalent vaccines.**** Data obtained from antigenic characterization are important in the assessment of the similarity between reference vaccine viruses and circulating viruses. In vitro antigenic characterization data acquired through hemagglutination inhibition (HI) assays or virus neutralization-based focus reduction assays (FRAs) evaluate whether genetic changes in circulating viruses affect antigenicity; substantial differences could affect vaccine effectiveness. Nearly all influenza viruses received by CDC are genomically characterized using next generation sequencing, and the genomic data are analyzed and submitted to public databases (GenBank: https://www.ncbi.nlm.nih.gov/genbank or EpiFlu: https://www.gisaid.org/). CDC antigenically or genetically characterized 680 influenza viruses collected and submitted by U.S. laboratories and laboratories outside the United States during May 20–October 13, including 351 influenza A(H1N1)pdm09 viruses, 185 influenza A(H3N2) viruses, and 144 influenza B viruses.

**FIGURE 3:**
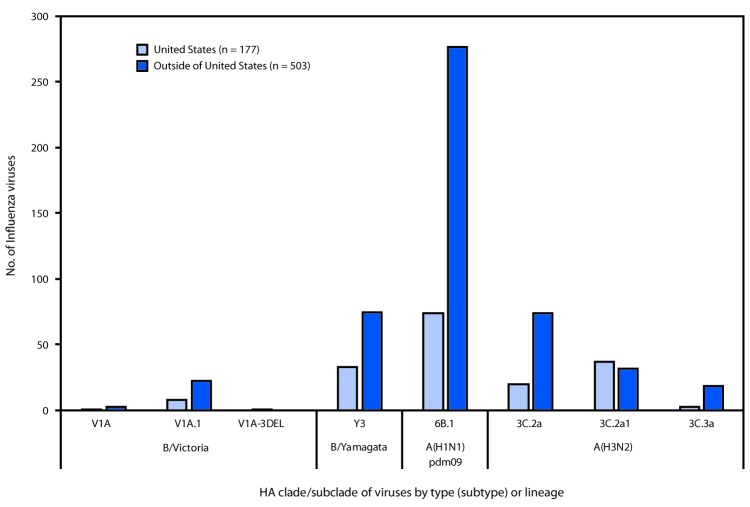
Genetic characterization of influenza viruses collected in and outside of the United States during May 20–October 13, 2018

Phylogenetic analysis of the A(H1N1)pdm09 hemagglutinin (HA) genes of viruses collected globally since May 20, 2018, identified viruses belonging to HA genetic subgroup 6B.1 (Figure 3). All of the A(H1N1)pdm09 viruses tested (57 in and 87 outside the United States) were antigenically similar (analyzed using HI tests with ferret antisera) to egg and cell-propagated A/Michigan/45/2015 viruses, which are in genetic group 6B.1 and are reference viruses representing the influenza A(H1N1) component of the Northern Hemisphere 2018–19 influenza vaccine.

Among 185 influenza A(H3N2) viruses collected and sequenced since May 20, 2018, phylogenetic analyses indicated cocirculation of multiple clades and subgroups of HA genes. The HA genes of the viruses belonged to genetic groups 3C.2a, 3C.2a1, and 3C.3a, with 3C.2a predominating (Figure 3). The majority of genetic group 3C.2a viruses (89% [86/94]) belonged to subclade 3C.2a2. A subset of 111 influenza A(H3N2) viruses was antigenically characterized by HI or FRA (43 in and 68 outside the United States); 102 (91.9%) were well inhibited by ferret antisera raised against cell-propagated A/Singapore/INFIMH-16–0019/2016 (3C.2a1), the reference virus representing the A(H3N2) component of Northern Hemisphere 2018–19 influenza vaccines. However, combined data generated by the WHO GISRS Collaborating Centers demonstrated that ferret antisera raised against egg-propagated A/Singapore/INFIMH-16–0019/2016-like viruses inhibited a smaller proportion of recently circulating viruses. In contrast, ferret antisera raised against egg-propagated A/Switzerland/8060/2017 inhibited the majority of viruses belonging to the globally-predominant subclade 3C.2a2, which was a factor leading to an update of the recommended influenza A(H3N2) component for egg-based vaccines for the 2019 Southern Hemisphere influenza vaccine.^††††^

Thirty-six influenza B/Victoria-lineage viruses were phylogenetically analyzed. All HA genes belonged to genetic group V1A and 31 (86.1%), belonged to subgroup V1A.1, represented by B/Colorado/06/2017, the reference virus representing the B/Victoria lineage component of Northern Hemisphere 2018–19 influenza vaccines. The V1A.1 subgroup is characterized by a two amino acid deletion in the HA at residues 162–163. One virus belonging to the genetic group V1A-3DEL was identified. This virus had a three amino acid deletion (amino acid residues 162–164) in the HA; similar viruses were identified sporadically in several countries in recent months. Eighteen of 19 antigenically characterized B/Victoria lineage viruses (10 in and nine outside the United States) were well inhibited by ferret antisera raised against cell-propagated B/Colorado/06/2017-like viruses.

Phylogenetic analysis of the influenza B/Yamagata lineage viruses sequenced showed that all HA genes belonged to genetic group Y3 (Figure 3). Among 65 influenza B/Yamagata lineage viruses antigenically characterized (32 in and 33 outside the United States), all were well inhibited by ferret antisera raised against cell-propagated B/Phuket/3073/2013-like viruses, the reference virus representing the influenza B/Yamagata lineage component of the Northern Hemisphere 2018–19 quadrivalent vaccines.

## Antiviral Resistance of Influenza Viruses

The WHO Collaborating Center for Surveillance, Epidemiology, and Control of Influenza at CDC tested 347 influenza virus specimens collected during May 20–October 13 from the United States and worldwide for resistance to oseltamivir, peramivir, and zanamivir, the influenza virus neuraminidase inhibitor antiviral medications currently approved for use against seasonal influenza. Among 134 influenza A(H1N1)pdm09 viruses (63 in and 71 outside the United States), 132 influenza A(H3N2) viruses (57 in and 75 outside the United States), and 81 influenza B viruses (44 in and 37 outside the United States) tested, all were susceptible to all three medications. High levels of resistance to the adamantanes (amantadine and rimantadine) persisted among influenza A(H1N1)pdm09 and influenza A(H3N2) viruses, which is consistent with the current recommendation to avoid use of these medications against influenza at this time.^§§§§^

## Composition of the 2019 Southern Hemisphere Influenza Vaccine

The WHO recommendations for influenza vaccine composition for the Southern Hemisphere 2019 season were made at the WHO Consultation and Information Meeting on the Composition of Influenza Virus Vaccines held September 24–27, 2018, in Atlanta, Georgia. The recommended components for the 2019 Southern Hemisphere egg-based influenza trivalent vaccines are an A/Michigan/45/2015 (H1N1)pdm09-like virus, an A/Switzerland/8060/2017 (H3N2)-like virus, and a B/Colorado/06/2017-like virus (B/Victoria lineage). For egg-based quadrivalent vaccines, an additional component, B/Phuket/3073/2013-like virus (B/Yamagata lineage), is recommended. It was recommended that the A(H3N2) component of non–egg-based vaccines be an A/Singapore/INFIMH-16–0019/2016-like virus. Compared with the composition of the 2018 Southern Hemisphere influenza vaccine formulation, these recommendations reflect an update to the influenza A(H3N2) component for egg-based vaccines, a change in the influenza B lineage included in the trivalent vaccine and a change in the influenza B/Victoria component. Compared with the composition of the Northern Hemisphere 2018–19 influenza vaccines, these recommendations reflect only one change, an update to the A(H3N2) component used in egg-based manufacturing. 

## Novel Influenza A Virus Infections

Fourteen human infections with novel influenza A viruses were reported in the United States during May 20–October 13. Influenza viruses that normally circulate in swine and not humans are called “variant” viruses when detected in humans and designated with the letter v after the subtype. One infection was associated with an influenza A(H3N2)v virus, and 13 were associated with influenza A(H1N2)v viruses. All but one infection occurred among persons aged <18 years. The A(H3N2)v virus infection was reported from Indiana in a patient who reported swine contact at an agricultural fair in the week before symptom onset. All A(H1N2)v virus infections were reported in August from three states: California (six cases), Ohio (four), and Michigan (three). Eleven of the 13 patients reported contact with swine at agricultural fairs, one reported attendance at an agricultural fair but no contact with swine, and one reported neither contact with swine nor attendance at an agricultural fair. Limited human-to-human transmission might have taken place with this last A(H1N2)v infection; however, no ongoing or sustained human-to-human transmission associated with any of these infections was identified. None of the novel influenza A virus infections resulted in hospitalization, and all patients recovered.

The genome of the one A(H3N2)v virus(A/Indiana/27/2018) was closely related to A(H3N2)v viruses detected during 2017 and viruses known to circulate in the U.S. swine population. Antigenic testing showed reduced inhibition by ferret antisera raised to the nearest A(H3N2)v candidate vaccine virus (CVV), but postvaccination antisera from adults vaccinated with the 2017–18 influenza vaccine reacted with the virus at titers that were within fourfold of those against the homologous reference virus, A/Michigan/15/2014, representing the A(H3N2) component of the 2017–18 seasonal influenza vaccines. Postvaccination sera collected from children, however, had lower titers to this virus than to the A/Michigan/15/2014 homologous virus titer. These studies indicate that vaccination with the 2017–18 seasonal influenza vaccine might offer less protection against this A(H3N2)v virus for children than adults.

All of the A(H1N2)v viruses detected had HA gene segments from the delta 2 sublineage of the swine influenza virus H1 HA lineage. The HA and neuraminidase gene segments of these viruses were closely related to 2017 and 2018 A(H1N2) influenza viruses circulating in the U.S. swine population, including swine identified at the agricultural fairs attended by infected persons and viruses sporadically detected in previous A(H1N2)v zoonotic infections. Antigenic testing demonstrated that all of the 2018 A(H1N2)v viruses were well inhibited by ferret antisera raised to the nearest CVV. HI reactivity of pooled, child and adult postvaccination antisera from persons vaccinated with the 2017–18 vaccine was below the limit of detection for all viruses tested. These studies indicate that vaccine viruses specially developed to prevent A(H1N2)v virus infections would be protective; however, vaccination with the seasonal vaccine would not offer any protection.

## Discussion

In the United States, ILI activity remained below baseline levels during May 20–October 13, 2018; low levels of laboratory-confirmed influenza were reported as a result of a mix of influenza A and B. In the Southern Hemisphere, low levels of influenza activity were observed with a predominance of A(H1N1)pdm09 viruses. Analysis of available viruses suggests that minimal drift of viruses has occurred. 

Vaccination before the onset of influenza activity is the primary strategy to prevent influenza-associated illness and its potentially serious complications. A recent report indicated the high prevalence of influenza illnesses resulted in approximately 79,000 deaths and 960,000 hospitalizations during the 2017–18 influenza season (https://www.cdc.gov/flu/about/disease/burden.htm). Influenza vaccination prevents millions of medical visits, tens of thousands of hospitalizations, and thousands of deaths each year, even with vaccine effectiveness estimates in the range of 40%–60%. Health care providers should urge their patients to get vaccinated by the end of October, if they have not already been vaccinated. Vaccination efforts should continue throughout the influenza season.

In late September, WHO issued its recommendations for the 2019 Southern Hemisphere influenza vaccine. Surveillance has shown that there has been no significant evidence of antigenic drift among circulating A(H3N2) viruses since the selection of viruses for the 2018–19 Northern Hemisphere vaccines was made in February. However, the influenza A(H3N2) component for egg-based vaccines was updated to address genetic and antigenic changes that occur when A(H3N2) vaccine viruses are propagated in eggs. The A(H3N2) component was updated because sera against egg-propagated A/Switzerland/8060/2017 (H3N2) virus showed better reactivity with an increasing number of circulating A(H3N2) viruses than sera generated against egg-propagated A/Singapore/INFIMH-16-0019/2016. No changes were recommended for the A(H3N2) component of cell-manufactured or recombinant vaccines. It is difficult to predict which influenza virus will predominate or what the season will be like, but there will likely be cocirculation of influenza A(H1N1), A(H3N2), and B influenza viruses.

Annual influenza vaccination is the best method for preventing influenza infection and its potentially serious complications. In the United States, annual influenza vaccine is recommended for all persons aged ≥6 months who do not have a contraindication (*1*). Influenza vaccination has been shown to reduce the risk for influenza illness, and a growing body of evidence suggests that vaccination also reduces the risk for serious influenza outcomes that can result in hospitalization and even death. A CDC study in 2017 showed influenza vaccination reduced the risk for influenza-associated death by 51% among children with underlying high-risk medical conditions and by 65% among healthy children (*2*). Most recently, an August 2018 study showed that influenza vaccination lessened the risk for severe influenza among adults, including reducing the risk for hospitalization and admission to the intensive care unit, and also lessened severity of illness (https://www.cdc.gov/flu/spotlights/vaccine-reduces-risk-severe-illness.htm). These benefits are especially important for persons at high risk for serious influenza complications, including persons aged ≥65 years, children aged <5 years, pregnant women, and persons with certain underlying long-term medical conditions, including heart and lung disease, or diabetes.

Ideally, influenza vaccination should be administered before the start of community influenza activity. However, health care providers should continue to offer annual influenza vaccine to unvaccinated persons as long as influenza viruses continue to circulate. For the 2018–19 influenza season, multiple influenza vaccines are approved and recommended for use; there is no preferential recommendation for one influenza vaccine product over another for persons for whom more than one is suitable (*1*). Children aged 6 months–8 years require 2 doses of influenza vaccine administered ≥4 weeks apart if they have not received at least 2 doses of influenza vaccine before July 1, 2018 (*3*). Those who have previously received at least 2 doses before this date only require a single dose for 2018–19, even if the 2 previous doses were not received during the same or consecutive seasons (*1*). For the 2018–19 season, interim supply projections by manufacturers for the U.S. market range from 163 to 168 million doses of influenza vaccine.

Influenza antiviral medications can serve as a valuable adjunct to annual influenza vaccination. Early treatment with influenza antiviral medication is recommended for patients with confirmed or suspected influenza who have severe, complicated, or progressive illness; who require hospitalization; or who are at high risk for influenza-related complications.^¶¶¶¶^ Early treatment has been shown to decrease time to symptom improvement (*4*–*7*) and to reduce secondary complications associated with influenza (*8*,*9*). Providers should not delay treatment until test results become available because treatment is most effective when given early in the illness, especially within 48 hours of symptom onset (*10*). Providers should also not rely on less sensitive assays such as rapid antigen detection influenza diagnostic tests to inform treatment decisions (*10*).

During May 20–October 13, fewer human infections with variant viruses were reported compared with most previous seasons.***** Most of these variant viruses were influenza A(H1N2)v viruses, and A(H1N2) viruses have predominated in swine in some regions of the United States.^†††††^ All but two of the patients with variant virus infections reported swine exposure and attendance at an agricultural fair; one only attended an agricultural fair, and another reported neither swine exposure nor attendance at an agricultural fair. Vulnerable populations, especially young children and other persons at high risk for serious influenza complications, should avoid swine barns at agricultural fairs or close contact with swine. Health care providers should consider novel influenza virus infections in persons with ILI and swine or poultry exposure, or with severe acute respiratory infection after travel to areas where avian influenza viruses have been detected.

Influenza surveillance reports for the United States are posted online weekly and are available at https://www.cdc.gov/flu/weekly. Additional information regarding influenza viruses, influenza surveillance, influenza vaccines, influenza antiviral medications, and novel influenza A virus infections in humans is available at https://www.cdc.gov/flu.

**Summary**

**What is already known about this topic?**

CDC compiles, collects, and analyzes data on influenza activity to monitor the timing and severity of each influenza season.

**What is added by this report?**

Reported Southern Hemisphere influenza activity was relatively low and fairly mild, with influenza A(H1N1)pdm09 viruses predominating in most regions. In the United States, influenza activity was low in all regions, typical for this time of year. Fourteen influenza variant virus infections were reported in the United States, and most were associated with exposure to swine.

**What are the implications for public health practice?**

Annual influenza vaccination is recommended for all persons aged ≥6 months who do not have a contraindication. Providers should encourage influenza vaccination now prior to the increase of influenza activity.

## Footnotes

* Data as of October 19, 2018.

† Influenza viruses that circulate in swine are called swine influenza viruses when isolated from swine, but are called variant influenza viruses when isolated from humans. Seasonal influenza viruses that circulate worldwide in the human population have important antigenic and genetic differences from influenza viruses circulating in swine.

§ https://www.cdc.gov/flu/swineflu/variant/preventspreadfactsheet.htm.

¶ The U.S. influenza surveillance system collects five categories of information from eight data sources: 1) viral surveillance (U.S. World Health Organization collaborating laboratories, the National Respiratory and Enteric Virus Surveillance System, and novel influenza A virus case reporting); 2) outpatient illness surveillance (U.S. Outpatient Influenza-Like Illness Surveillance Network); 3) mortality (the National Center for Health Statistics Mortality Surveillance System and influenza-associated pediatric mortality reports); 4) hospitalizations (FluSurv-NET, which includes the Emerging Infections Program and surveillance in three additional states); and 5) summary of the geographic spread of influenza (state and territorial epidemiologist reports). https://www.cdc.gov/flu/weekly/overview.htm.

** Defined as a fever (temperature ≥100°F [≥37.8°C]), oral or equivalent, and cough and/or sore throat, without a known cause other than influenza.

†† The national and regional baselines are the mean percentage of visits for influenza-like illness (ILI) during noninfluenza weeks for the previous three seasons plus two standard deviations. Noninfluenza weeks are defined as periods of ≥2 consecutive weeks in which each week accounted for <2% of the season’s total number of specimens that tested positive for influenza in public health laboratories. National and regional percentages of patient visits for ILI are weighted based on state population. Use of the national baseline for regional data are not appropriate.

*** The seasonal baseline proportion of pneumonia and influenza (P&I) deaths is projected using a robust regression procedure, in which a periodic regression model is applied to the observed percentage of deaths from P&I that were reported by the National Center for Health Statistics Mortality Surveillance System during the preceding 5 years. The epidemic threshold is set at 1.645 standard deviations above the seasonal baseline.

††† http://www.who.int/influenza/gisrs_laboratory/collaborating_centres/en.

§§§ In temperate climates, the onset and peak of influenza activity might vary substantially from one influenza season to the next, but generally begins to increase in the late fall. In the Northern Hemisphere’s temperate regions, annual epidemics of influenza typically occur during October–February, but the peak of influenza activity can occur as late as April or May. In temperate regions of the Southern Hemisphere, influenza activity typically peaks during May through August. Although temperate regions of the world experience a seasonal peak in influenza activity, influenza viruses can be isolated year-round. The timing of seasonal peaks in influenza activity in tropical and subtropical countries varies by region. Multiple peaks of activity during the same year have been seen in some areas and influenza infection can occur year-round.

¶¶¶ http://www.who.int/influenza/surveillance_monitoring/updates/en/.

**** http://www.who.int/influenza/vaccines/virus/recommendations/2018_19_north/en/.

†††† http://www.who.int/influenza/vaccines/virus/recommendations/201809_recommendation_report.pdf?ua.

§§§§ https://www.cdc.gov/flu/professionals/antivirals/links.htm.

¶¶¶¶ Persons at high risk include 1) children aged <2 years; 2) adults aged ≥65 years; 3) persons with chronic pulmonary conditions (including asthma), cardiovascular disease (except hypertension alone), renal, hepatic, hematologic (including sickle cell) disease, metabolic disorders (including diabetes mellitus), or neurologic and neurodevelopmental conditions (including disorders of the brain, spinal cord, peripheral nerves, and muscles, such as cerebral palsy, epilepsy [seizure disorders], stroke intellectual disability [mental retardation], moderate to severe developmental delay, muscular dystrophy, or spinal cord injury); 4) persons with immunosuppression, including that caused by medications or by human immunodeficiency virus infection; 5) women who are pregnant or postpartum (within 2 weeks after delivery); 6) persons aged ≤18 years who are receiving long-term aspirin therapy; 7) American Indians/Alaska Natives; 8) persons with extreme obesity (i.e., body mass index ≥40); and 9) residents of nursing homes and other chronic care facilities.

***** https://gis.cdc.gov/grasp/fluview/Novel_Influenza.html.

††††† https://www.aphis.usda.gov/animal_health/animal_dis_spec/swine/downloads/fy2018quarter1swinereport.pdf.
